# Foreign body aspiration through tracheotomy: a case report

**DOI:** 10.1016/S1808-8694(15)31316-1

**Published:** 2015-10-20

**Authors:** Ricardo R. Figueiredo, Walter S. Machado

**Affiliations:** 1 Otorhinolaryngologist, Hospital Municipal Souza Aguiar, Rio de Janeiro; 2 Head of the Service of Otorhinolaryngology and Perioral Endoscopy, Hospital Municipal Souza Aguiar, Rio de Janeiro

**Keywords:** bronchus, foreign bodies, bronchoscopy, tracheotomy

## Abstract

A 70 year-old man, with a 7-year tracheotomy because of a laryngeal tumor, had an accident during daily canulla cleansing procedure, aspirating a piece of the cleaning brush. Chest radiograph showed metallic foreign body at the right inferior bronchus. Rigid bronchoscopy was performed under general anesthesia, with no resistance in passing the tube through the glottis. The foreign body was easily removed and the patient had no complications. After leaving the hospital, the patient was sent to the ENT service where he used to be followed up.

## INTRODUCTION

Foreign bodies in the tracheobrochial tree correspond to about 0.073% of cases seen at the Service of Otorhinolaryngology and Perioral Endoscopy, Hospital Souza Aguiar, Rio de Janeiro. However, they are the ones that provide more risk, owing to higher incidence of complications, including death, especially in those cases in which intervention takes longer.

Aspiration of foreign bodies through tracheostomy orifices is an uncommon occurrence, and there are few references in the literature. There are more difficulties detected when it is impossible to pass the bronchoscope through glottic space and it may be necessary to resort to flexible bronchoscopy, rarely effective for removal of foreign bodies, leading eventually to surgical removal by thoracotomy.

## CASE REPORT

**Figure 1:**
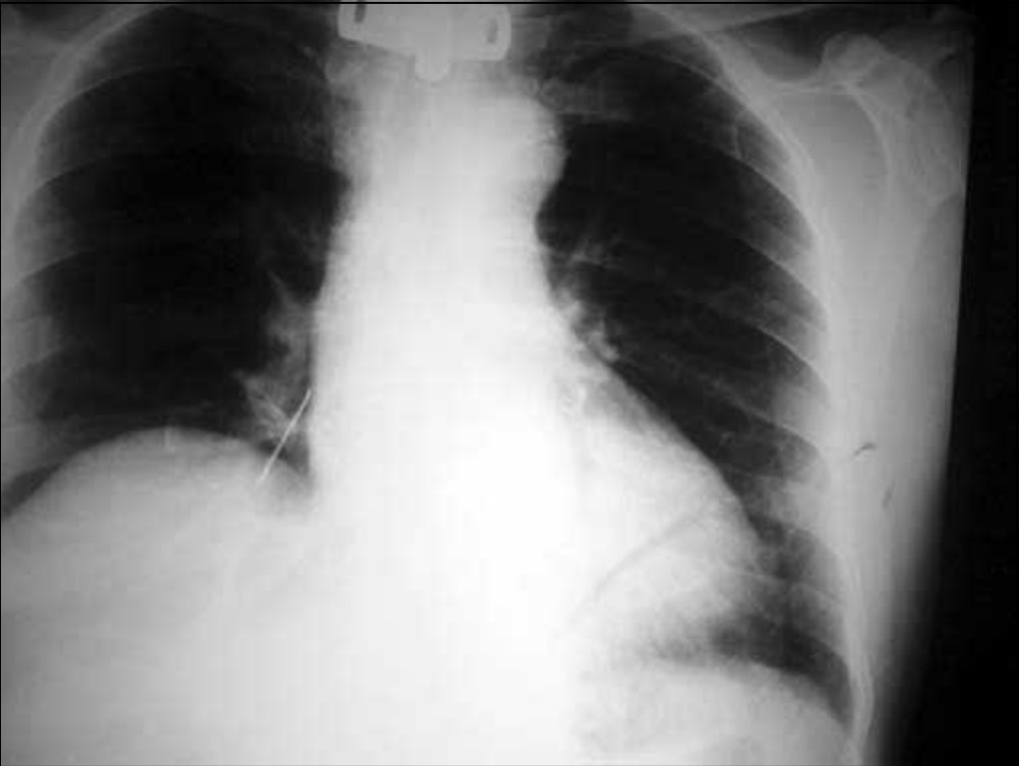
Chest X-ray in PA showing foreign body in right source bronchus.

**Figure 2:**
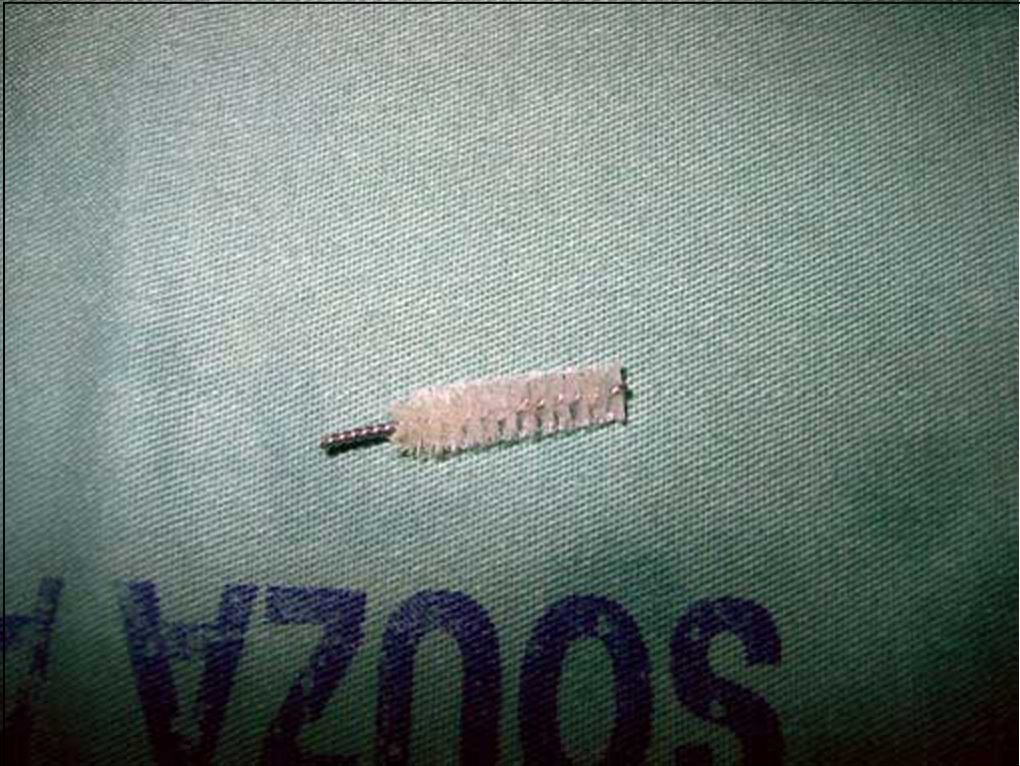
Bronchial foreign body (metallic brush to clean the tracheotomy canulla).

E.G.S., male, 70 years old, came to our hospital on 09/01/2003 with history of aspiration through tracheotomy canulla of a fragment of the cleaning brush, some hours before. The brush had a metallic portion recovered by plastic brittles. The patient had been tracheotomized for 7 years owing to laryngeal tumor. Indirect laryngoscopy with optical fiber evidenced moderate edema of vocal folds with no signs of tumor. The patient was eupneic, with cough and discharge of small amount of mucoid secretion. Chest x-ray in PA (Figure 1) showed images of the metallic object in the right lower bronchus, no signs of associated atelectasia or hyperinsuflation. We decided for immediate rigid bronchoscopy under general anesthesia. The passage of the tube through glottic space happened uneventfully, and we removed the tracheotomy canulla. The foreign body was located in the right inferior bronchus and we easily removed it (Figure 2). Bronchoscope was removed and orotracheal intubation was made by the anesthesiologist, and the canulla was replaced. Postoperative evolution was excellent and he was discharged the next day. We prescribe cephalexine and instructed the patient to go back to the center where he had underwent tracheotomy to check the need for a new procedure.

## DISCUSSION

Foreign bodies in the tracheobronchial tree are rare occurrences, more common in pediatric emergency rooms. At Hospital Souza Aguiar, they represent about 0.073% of the visits to the Service of Otorhinolaryngology and Perioral Endoscopy. The reason for a relatively small percentage (about 0.60% of foreign bodies) is a result of the protection of airways with epiglottis, arytenoids and coughing reflex. The main sites for impaction are, in order, right source bronchus, left source bronchus and trachea^1^, ^2^, ^3^

According to the literature^1^, ^2^, ^3^, ^4^, ^5^, most of the cases happen up to 3 years of age, with slight predominance of male. The most frequent aspirated objects are seeds, including, among others, beans, rice and peanuts, and small metal and plastic objects, such as fragments or parts of toys.

We should always try to find out about the suggestive history of foreign body aspiration, such as for example, ask the child whether he/she had been eating or playing with some small object, if there was strong cough or cyanosis. For adults, history is normally evident, accidents normally occur during the meals, with fish spines, grains or chicken bones that may be aspirated. In our service, we have had uncommon cases, such as the patient that aspirated ammunition when he was embraced by his daughter while cleaning the gun and holding the bullet in his mouth. However, the history is not always clear and if in doubt, we should always perform exploratory bronchoscopy^1^, ^2^, ^3^, ^4^.

Clinical picture is normally characterized by cough, wheezing, snoring, dyspnea and tracheal symptoms, if the foreign body is located in the trachea. The patient may present fever and mucus-purulent expectoration in the case of associated pneumonia and progressive dyspnea, which may get as bad as respiratory failure. Pulmonary auscultation may reveal wheezing, snoring and reduction of vesicular murmur of affected area. Wheezing and snoring in patients without previous history of bronchial asthma should be warning signs. Suprasternal draught may be present in cases of respiratory failure, as well as cyanosis in more extreme cases^1^, ^3^, ^4^.

The main complementary exam is simple chest x-ray in PA, in which the 3 main findings are:

• image of foreign body, if radiopaque;
• suggestive images of atelectasia, such as deviation of mediastinum and diaphragm;
• images suggestive of emphysema by vascular mechanism, such as hyperinsuflation.

Once there is diagnostic suspicion, we should immediately proceed with rigid bronchoscopy under general anesthesia. The literature is unanimous to say that complications normally take place when it takes too long to intervene, normally because of diagnostic uncertainty^1^, ^3^, ^4^, ^5^. The complications include pneumonia, pneumo-mediastinum, pneumothorax, mediastinitis, respiratory failure and death^1^, ^6^. Bronchoscopy is a relatively simple procedure in most cases, and we should emphasize the need of appropriate training practice and the right material (rigid bronchoscope of different sizes and clamps for bronchial foreign bodies)^7^, ^8^.

Foreign bodies in tracheobronchial tree aspirated through tracheotomy are very rare, and there are few reports in the literature, some associated with psychiatric disorders, with repetitive introduction of varied objects into the tracheostome^9^. Our patient presented the necessary conditions to allow passage of bronchoscope through glottic access, maybe because he no longer needed the tracheotomy. This fact has really facilitated the procedure. In laryngectomized patients^7^, ^10^, and in all those in which it is not possible to pass the bronchoscope through the larynx, it may be necessary to resort to surgical procedure^11^, given that it is quite difficult to allow passage of rigid bronchoscope through the tracheotomy orifice, including the risk of marked tracheal bleeding owing to friction. Flexible bronchoscope is rarely efficient to remove foreign bodies, but we believe that an attempt should always be made before the surgical procedure.

## CONCLUSION

Foreign bodies in the tracheobronchial tree are rare occurrences in emergencies, and they are more frequent in children. Diagnosis is clinical and radiological, and intervention should be as quickly as possible using rigid bronchoscopy. Most complications, including death, are resultant from delay in performing the procedure. Aspiration of foreign body through tracheostome is an extremely rare event, and it may require surgical intervention for its removal.
